# The prevalence and risk of Female Genital Mutilation/Cutting among migrant women and girls in the Netherlands: An extrapolation method

**DOI:** 10.1371/journal.pone.0230919

**Published:** 2020-04-09

**Authors:** Ramin Kawous, Maria E. T. C. van den Muijsenbergh, Diana Geraci, Anke van der Kwaak, Els Leye, Annemarie Middelburg, Livia E. Ortensi, Alex Burdorf

**Affiliations:** 1 Department of Public Health, Erasmus University Medical Center, Rotterdam, The Netherlands; 2 Pharos, Dutch Centre of Expertise on Health Disparities, Utrecht, The Netherlands; 3 Department of Primary and Community care, Radboud University Medical Centre Nijmegen, Nijmegen, The Netherlands; 4 Department of Development Policy and Practice, Royal Tropical Institute, Amsterdam, The Netherlands; 5 International Centre for Reproductive Health, Faculty of Medicine, Ghent University, Ghent, Belgium; 6 Middelburg Human Rights Law Consultancy, Maarssen, The Netherlands; 7 Department of Statistical Sciences "Paolo Fortunati", Alma Mater Studiorum—University of Bologna, Bologna, Italy; Hackensack University Medical Center, UNITED STATES

## Abstract

**Objectives:**

The aim of the study was (I) to estimate the prevalence of Female Genital Mutilation/Cutting (FGM/C) and distribution of types of FGM/C among migrant girls and women in the Netherlands, and (II) to estimate the number of migrant girls at risk of being cut in the immediate future.

**Methods:**

National population-based survey data regarding FGM/C prevalence were applied to female migrants in the Netherlands who migrated from 29 countries with available nationally representative data on FGM/C.

**Results:**

As of January 1^st^ 2018, there were 95,588 female migrants residing in the Netherlands, originating from 29 countries with available nationally representative data on FGM/C. Our findings suggest that about 41,000 women had undergone FGM/C, of which 37% had Type III (infibulation). In total 4,190 girls are estimated to be at risk of FGM/C in the next 20 years, of whom 394 were first-generation girls.

**Conclusion:**

These findings show the urgency to develop appropriate strategies and policies to prevent FGM/C, to protect girls and women at risk of the practice, and to provide adequate services and support for those affected by FGM/C in the Netherlands.

## Background

Female Genital Mutilation/Cutting (FGM/C) ‘comprises all procedures involving the partial or total removal of the external female genitalia, or other injury to the female genital organs for non-medical reasons’ [1]. The World Health Organization classifies FGM/C into four types, depending on the extent of cutting, ranging from a nick of the clitoris to infibulation, which involves the removal of clitoris and labia with the remaining genitals sewing up, leaving only a small opening [1]. FGM/C, especially infibulation, has been associated with an increased risk of health complications, including severe pain, excessive bleeding, urinary tract infections, bacterial vaginosis, painful sexual intercourse and adverse perinatal outcomes [2–4] as well as negative effects on mental health [5–7].

FGM/C is practiced predominantly in countries in Africa, the Middle East and Asia. The prevalence of FGM/C is routinely measured through the Demographic and Health Survey (DHS) and the Multiple Indicator Cluster Survey (MICS). The United Nations Children’s Fund estimates that in over 30 countries at least 200 million girls and women alive today have undergone FGM/C [8] and, according to the United Nations Population Fund about 68 million girls are at risk of being cut between 2015–2030 [9]. As a consequence of migration from countries where FGM/C is concentrated, the practice has become a global issue and a rising concern in Western countries. In order to develop effective interventions to prevent FGM/C from being practiced in Western countries and to provide appropriate health care to girls and women who have undergone FGM/C, it is important to have insight into the prevalence of FGM/C in countries of destination. Therefore, the European parliament has called for better data and methods to estimate the number of girls and women who have undergone FGM/C and girls at risk for the practice in Europe [10,11].

A population-based survey in countries of destination to determine the prevalence of FGM/C in migrant communities, similar to DHS and MICS, is challenging due to, among others, financial constraints and methodological issues. Therefore, most estimates of FGM/C among female migrants in Western countries are based on extrapolation from DHS and MICS prevalence data on FGM/C from countries of origin to the resident migrant populations [12].

Using this model for indirect estimation, it was estimated in 2012 that about 29,000 girls and women in the Netherlands were already subjected to FGM/C, and that between 600 and 3,800 girls were at risk of FGM/C in the next 15 years in the Netherlands [13].

Studies on the prevalence- and risk of FGM/C among migrant women and girls in European countries have been challenged, due to, among others, several methodological constraints in the field, including, for example, the use of national FGM/C prevalence [12]. One limitation of using national FGM/C prevalence is that it does not account for variation in FGM/C prevalence across regions and different ethnicities [12–14]. Despite some of these methodological issues, recent studies in the field of indirect estimation have improved their estimates by taking into account, for example, differences between first- and second-generation migrants, ages at the time of arrival, places of birth, and the selection process of migrants [13,15–18]. Other improvements could be adopted, for instance, to adjust FGM/C estimates for time-dependent patterns of the practice in country of origin, age distributions before and after migration, impact of migration and acculturation, and enforcement of preventive measures to target girls potentially at risk in country of destination, which will affect first- and second-generation female migrants differently.

The introduction of actively enforced preventive measures to target girls potentially at risk in country of destination is of particular interest. In 1993, an official statement in the Netherlands was made declaring that FGM/C was prohibited under criminal law. However, an active prevention and prosecution policy with nationwide coverage was not implemented until 2006 [19–21]. The rationale behind the Dutch approach against FGM/C is to prevent FGM/C by accomplishing a behavioural change towards the practice. The prevention policies in the Netherlands consist of, among others, awareness raising on FGM/C among communities concerned and the empowerment of women, as well as training of health professionals and health care workers to support women affected by FGM/C and to identify and prevent girls from being cut [22]. To our knowledge, no prevalence study has assessed the influence of preventive measures to target girls potentially at risk in country of destination on FGM/C estimates.

The aim of present study was (I) to estimate the prevalence of FGM/C and distribution of types of FGM/C among female migrants in the Netherlands, and (II) to estimate the number of girls at risk of being cut in the immediate future, with age-and region-specific estimates for periods before- and after migration. These estimates were adjusted to account for the effect of ‘*migration and acculturation*’ and ‘*preventive measures’* to target girls potentially at risk in country of destination.

## Methods

### Study population

The study population consisted of first- (*n* = 60,297) and second-generation female migrants (*n* = 35,291) residing in the Netherlands at January 1^st^, 2018 (reference year/date), originating from the 29 countries with available nationally representative data on FGM/C (see Table 1).

First-generation migrants refer to girls and women who migrated from these countries, whereas second-generation migrants refer to girls born in the Netherlands to at least one parent who has migrated from one of these countries.

**Table 1:** DHS and MICS data: Country of origin, source- and year of publication, overall prevalence (%) and the prevalence of Type III and other types of FGM/C (%); Statistics Netherlands dataset on first- and second-generation female migrants.

Country of origin	DHS and MICS data					Statistics Netherlands		
** **	Source	Year of publication	Overall FGM/C prevalence (%)	Type of FGM/C (%)		First-generation	Second- generation	Total
				Type III	Other types or unknown			
Benin	MICS	2014	9.2	10.1	89.9	102	83	185
Burkina Faso	DHS	2010	75.8	1.2	98.8	135	145	280
Cameroon	DHS	2004	1.5	5.0	95.0	924	697	1621
Central African Republic	MICS	2010	24.3	7.0	93.0	30	10	40
Chad	DHS	2014–15	38.4	9.4	90.6	27	39	66
Côte d'Ivoire	DHS	2011–12	38.2	8.7	91.3	530	351	881
Djibouti	MICS	2006	93.2	67.2	32.8	79	63	142
Egypt	DHS	2015	87.2	0.7	99.3	4716	5214	9930
Eritrea	PHS	2010	83.0	38.6	61.4	6271	784	7055
Ethiopia	DHS	2016	65.2	6.5	93.5	7266	2920	10186
Gambia	DHS	2013	74.9	0.0	100.0	347	286	633
Ghana	MICS	2011	3.8	7.9	92.1	7255	4864	12119
Guinea	DHS	2012	96.9	7.5	92.5	1118	979	2097
Guinea-Bissau	MICS	2014	44.9	6.0	94.0	106	78	184
Iraq	MICS	2011	8.1	0.0	100.0	7004	3038	10042
Kenya	DHS	2014	21.0	9.3	90.7	1546	944	2490
Liberia	DHS	2013	49.8	0.0	99.9	593	692	1285
Mali	DHS	2012–13	91.4	10.6	89.4	82	95	177
Mauritania	MICS	2015	66.6	4.5	95.5	32	62	94
Niger	DHS	2012	2.0	6.3	93.7	54	74	128
Nigeria	DHS	2013	24.8	5.3	94.7	3038	3025	6063
Senegal	DHS	2016	22.7	7.1	92.9	385	419	804
Sierra Leone	DHS	2013	89.6	9.0	91.0	1349	973	2322
Somalia	MICS	2006	97.9	79.3	20.7	12924	6824	19748
Sudan	MICS	2014	86.6	77.0	23.0	1909	1101	3010
United Republic of Tanzania	DHS	2015–16	10.0	6.6	93.4	565	606	1171
Togo	DHS	2013–14	4.7	15.4	84.6	446	377	823
Uganda	DHS	2016	0.3	0.0	100.0	1104	411	1515
Yemen	DHS	2013	18.5	0.0	100.0	360	137	497
**Total**	** **	** **	** **	** **	** **	**60297**	**35291**	**95588**

The Netherlands has a large number of migrants from Indonesia. Although FGM/C is practised in Indonesia, we did not include female migrants from Indonesia in the present study. Nationally representative data on FGM/C in Indonesia is available only for girls under the age of 11 years. This data shows that nearly half of girls (49%) under the age of 11 have undergone some form of FGM/C [23]. We deemed it not reasonable to assume a similar prevalence among girls and women above 12 years.

For this study, Statistics Netherlands (CBS) provided microdata on first- and second-generation female migrants at January 1^st^, 2018 by country of origin, birth place, date of arrival in the Netherlands, and current age. Under the General Data Protection regulation these indirectly identifiable data are available for scientific research under strict conditions of confidentially and ethical requirements [24]. Using Microsoft office Excel (2016) and IBM SPSS Statistics (version 25.0), the dataset had to be processed by regrouping the female migrants according to regions within their countries of origin and calculating their age upon arrival in the Netherlands. We also received access to data from Central Agency for the Reception of Asylum Seekers (COA) about female asylum seekers in the reception centres by current age and country of origin.

### FGM/C practice in countries of origin

The most recent DHS and MICS were provided by ICF International and UNICEF on September 12, 2017 and September 7, 2017 respectively (see Table 1). DHS and MICS are nationally representative household surveys, covering a range of demographic and health issues in developing countries including FGM/C. Since DHS and MICS surveys are limited to women of reproductive age (15 to 49), assumptions were made on FGM/C prevalence for age groups under 15 and above 49.

Data on the prevalence of FGM/C among daughters aged 0 to 14 as reported by their mothers are available. Since many of these girls may not have reached the customary age at the time of the survey, prevalence data for girls under 15 reflect their current, however not their final FGM/C status [14]. We therefore used data on age at which FGM/C is performed, derived from respondents between 15 and 49 years in DHS and MICS surveys. These data were first adjusted for women with missing information on age at cutting, and subsequently age-specific proportions were multiplied with the national FGM/C prevalence for age group 15 to 19 years. This procedure provided for each country of interest an age-specific prevalence of FGM/C for the 5 years age groups 0–4, 5–9, and 10–14. For women older than 49 years of age, we assumed that they would have the same FGM/C prevalence as women aged 45 to 49, the oldest age group for which information was available in the DHS and MICS surveys. The age-specific prevalence across age groups captures the increase in new cases of FGM/C in each older age group. This procedure will present FGM/C estimates with much higher precision than using a dichotomous estimate based on median age of cutting or relying on the FGM/C prevalence in the age group 15–19 only.

For countries with substantial regional variation in FGM/C, the age-specific prevalence per region was calculated, based on regional distribution of FGM/C in the most recent survey. For 8 countries with a national FGM/C prevalence well above 80% and small regional differences (see Table 1), this adjustment was not applied. A similar approach was considered for ethnicity, but since information on ethnicity of migrants in the Netherlands was not available no further stratification on FGM/C prevalence was used.

### Estimation of FGM/C occurrence among female migrants in the Netherlands

#### Migration and acculturation and preventive measures in country of destination

Similar to the present study, previous studies have taken into consideration factors in the extrapolation model on the number of girls at risk, that capture important differences between country of destination and country of origin [25]. The first impact factor relates to migration and acculturation. Through migration it is expected that some acculturation will take place, referring to “the dual process of cultural and psychological change that takes places as a result of contact between two or more cultural groups and their individual members” [26]. There is no empirical evidence available, but it is a reasonable assumption that FGM/C practice will be less common among migrants with permanent residence in the Netherlands, than in their country of origin. Hence, the impact of “migration and acculturation” can be introduced as a reduction factor on FGM/C estimates with a value between 0 (perfect adoption of Dutch culture with respect to FGM/C) and 1 (no influence whatsoever on FGM/C practice).

The second impact factor relates to ‘preventive measures’ in the country of destination. In the Netherlands, an active prevention policy with nationwide coverage was implemented in 2006 [19–21]. In the Netherlands, at set ages from birth onwards, health and development of children between 0 and 19 years of age are monitored by Youth Health Care centres. These centres play an important role in providing health promotions and disease prevention, among which FGM/C [27]. Youth health care professionals are required to inform parents from countries where FGM/C is practiced that FGM/C is prohibited and prosecution could follow if FGM/C is established. These professionals are also required to identify and assess the risk of FGM/C [21]. In this study, a reduction factor was introduced to adjust the risk estimation from the year 2006 onward, in order to account for the influence of preventive measures. The challenge would be to assess the relative change in attitude towards discontinuation of FGM/C among migrants, and to assess the extent to which preventive measures may have been effective. This challenge arises from the lack of empirical data that capture change in the practice of FGM/C after migration.

Therefore, in the absence of such data, we performed a sensitivity analysis in which we varied the impact factors, including a scenario in which no reduction was applied, that is, no corrections were made to take into account the influence of migration and acculturation and preventive measures on risk estimation. The sensitivity analysis was conducted with arbitrary reduction factors ranging from 0% to 100%. The procedure provided lower- and upper boundaries for the reported estimates on FGM/C prevalence- and risk, but these boundaries cannot be regarded as confidence intervals. However, the no reduction scenario, that is maximum estimate of the number of girls at risk of being cut, would imply that migrants have not been acculturated towards discontinuation of FGM/C, while there is increasing evidence on attitude change toward discontinuation of the practice in destination countries that indicate FGM/C is practiced at lower rates than in countries of origin [28–32]. This scenario would also imply that preventive measures in the Netherlands have not been effective at all. There is no indication of FGM/C being performed in the Netherlands and no suspected FGM/C case has, as yet, been convicted by a court [33]. It is therefore safe to attribute this to the preventive measures in the Netherlands which target girls potentially at risk of FGM/C. The 50:50% reduction was considered the neutral scenario. We acknowledge that this scenario is speculative, but in the absence of data it rather indicates an equal balance between the preventive measures being effective and not being effective, and an equal balance between migrants acculturating towards discontinuation of the practice and not acculturating towards discontinuation of the practice.

#### FGM/C prevalence- and risk estimation

In compliance with earlier studies (e.g., ref. [15]) girls and women were categorized in those hypothesized to have been cut before their migration to the Netherlands, those hypothesized to have been cut after their migration, and those hypothesized to have not been cut, but potentially still are at risk of the practice.

Since FGM/C has a strong positive social and cultural value, the practice may continue after migration. While many re-evaluate and abandon FGM/C in exile [28–32], there are some indications that FGM/C is practised in Europe (e.g., France, Italy, Switzerland and United Kingdom) by traditional practitioners or by medical personnel [17,34,35]. In addition, there is emerging evidence indicating that girls undergo FGM/C during their holiday in countries of origin [36,37]. We therefore assumed that at a certain point in time, some girls born in the Netherlands, and some girls who at the time of arrival in the Netherlands were younger than 19, have undergone FGM/C after their migration to the Netherlands.

In order to avoid underestimation, in the present study, we assumed a maximum age of cutting of 20 years, as in some countries girls may still be at risk of undergoing cutting after the age of 15 [11,38]. The estimation method accounted for time-varying patterns of FGM/C practice during different periods of calendar time and ageing of individuals in country of origin and in the Netherlands.

**Fig 1:**
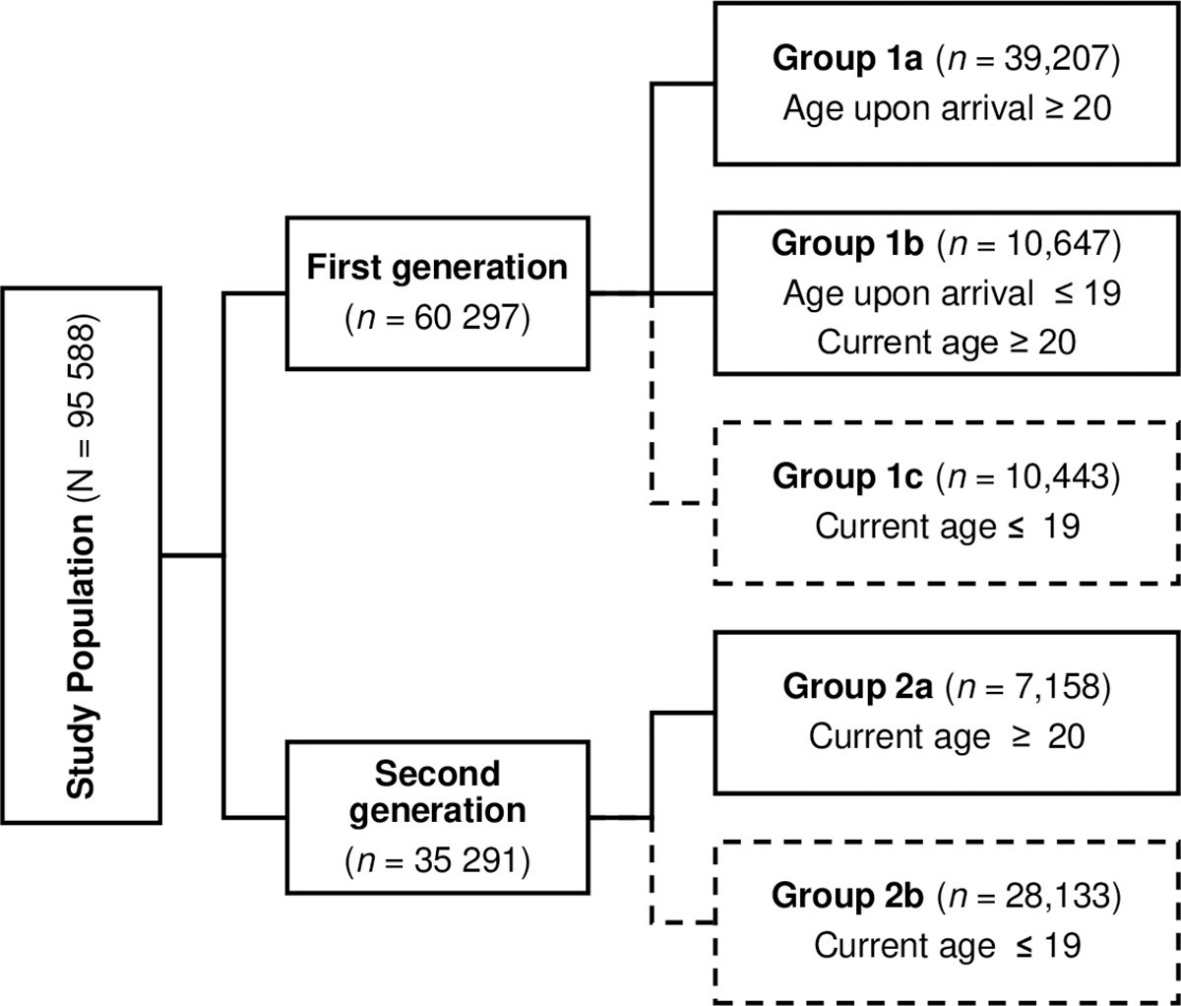
Distinction within female migrants in the Netherlands. Groups that only contribute to the prevalence of FGM/C at January 1st, 2018 (solid boxes) and groups that contribute both to the prevalence of FGM/C and to potential new cases thereafter (dashed boxes).

As shown in Fig 1, female migrants were further divided into first- and second-generation. The first-generation girls and women consisted of three groups (groups 1a, 1b, and 1c), based on age at arrival and age at January 1^st^, 2018. The second-generation girls and women consisted of only two groups (groups 2a, and 2b), based on age at January 1^st^, 2018. Those who had reached the age of 20 at the reference date (groups 1a, 1b, and 2a) were included in the estimation of FGM/C prevalence and regarded as no longer at risk for FGM/C. The younger female migrants (groups 1c, 2b) comprised girls below age of 20, who may have been cut before the reference date or who may be still at risk for FGM/C after the reference date. Hence, these latter groups contributed to both the FGM/C prevalence as well as the FGM/C risk.

Group 1a comprised girls and women who may have been subjected to FGM/C before their migration to the Netherlands at age 20 or older. After arrival in the Netherlands, new cases of FGM/C were considered not possible. The prevalence of FGM/C in this group was obtained by applying the age-specific FGM/C prevalence in the country of origin, adjusted for regional differences in FGM/C prevalence when necessary, to the age composition of female migrants in the Netherlands.

Group 1b consisted of girls and women who at the time of arrival in the Netherlands were 19 years or younger, and who were 20 years or older at January 1^st^, 2018. The prevalence of FGM/C in this group was calculated in two steps. First, we applied the age-specific FGM/C prevalence of region in country of origin to all girls until their age of arrival in the Netherlands. In the second step, from that age until their 20^th^ birthday they were considered to be exposed to FGM/C practice among female migrants in the Netherlands.

In our model, we introduced a *'preventive impact factor’* of 50% on FGM/C practice in the Netherlands after January 1^st^ 2006, due to nationwide preventive measures to target girls potentially at risk as described below. Girls who reached the age of 20 years before 2006 were assigned the age-specific FGM/C prevalence according to their country- and region of origin. Girls still below this age at start of 2006 were assigned a 50% reduction factor on FGM/C for their remaining years from age at 2006 until age 20.

Group 1c comprised girls who at the time of arrival in the Netherlands were 19 years or younger and who were still 19 years or younger at January 1^st^, 2018. In line with Group 1b, we first applied the age-specific FGM/C prevalence of region and country of origin to all girls until their age of arrival in the Netherlands. After arrival in the Netherlands, for age years before 2006 the prevalence of FGM/C was determined by the same procedure as in the first step. For age years from 2006 onwards until 2018 we again assigned a 50% reduction factor to the estimation of the prevalence of FGM/C. At January 1^st^, 2018, we first estimated the population-at-risk, i.e. the number of girls still younger than 19 years minus those girls who had underwent FGM/C in the years before. Subsequently, for this population-at-risk the estimation of the number of girls potentially at risk for FGM/C after the reference date was based on 50% reduction factor on the age-specific FGM/C prevalence in country of origin.

Second-generation migrants were distinguished into two groups (groups 2a and 2b), depending on whether girls were still at risk for FGM/C after the reference date. We assumed that second-generation women aged 20 years or older at January 1^st^ 2018 (Group 2a) had a lower risk for FGM/C due to migration and acculturation on attitudes and behaviour towards cutting. For years lived before January 1^st^ 2006, the ‘*migration and acculturation’* impact factor was set at 50% for age-specific FGM/C prevalence from birth onwards. For years lived thereafter, the nationwide preventive measures to target girls potentially at risk in the Netherlands would also introduce a 50% reduction, and, thus, the combined effect of *‘migration and acculturation’ and ‘preventive measures’* to target girls potentially at risk was set at 75% reduction. Hence, age-specific FGM/C prevalence of regions within countries of origin with reduction factors of 50% and 75% were applied in this group, depending on years spent before and after the reference date in 2006, as described below.

The second-generation girls in Group 2b contributed to both the FGM/C prevalence at January 1^st^, 2018, as well as the risk for FGM/C in the near future. A similar procedure was followed as in Group 2a, with reduction factors on age-specific FGM/C prevalence of 50% or 75%, depending on years lived before and after January 1^st^ 2006. Since these girls were younger than 20 years of age at January 1^st^, 2018, this group also contributed to the FGM/C risk in the near future. A similar procedure was adopted as for Group 1c, albeit with a reduction factor of 75%.

#### Type of FGM/C occurrence among female migrants in the Netherlands

We estimated the proportion of those who have undergone infibulation, the most severe form of FGM/C (Type III), by extrapolating the distribution across different types of FGM/C in country of origin to our study population of female migrants in the Netherlands. Other types of FGM/C were merged into a single category called ‘Other types of FGM/C’.

## Results

**Fig 2:**
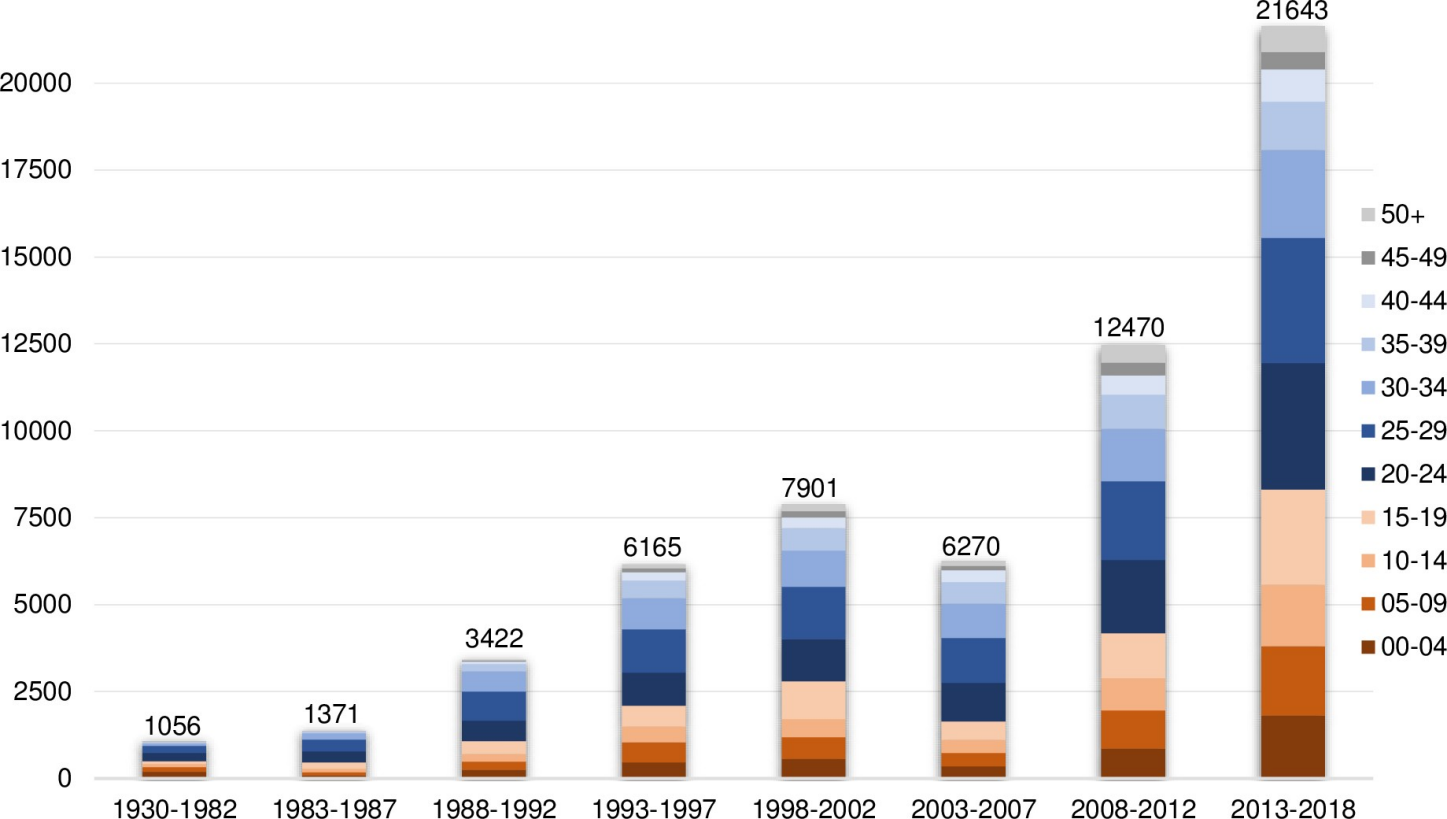
Number of first-generation girls and women by year of arrival and ages at the time of arrival in the Netherlands.

**Fig 3:**
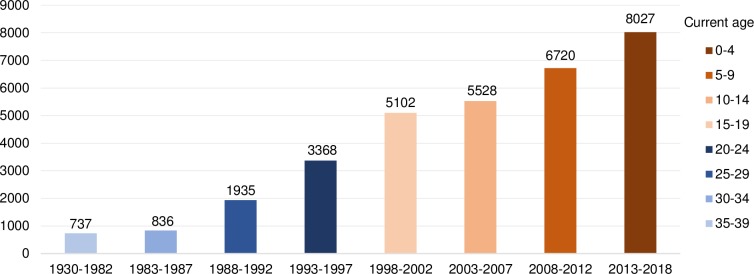
Number of second-generation girls and women by year of birth and current age.

As of January 1^st^ 2018, there were about 95,000 female migrants residing in the Netherlands whose country of origin is one of the 29 countries with available nationally representative data on FGM/C, of which 37% were second-generation girls and women (see Figs 2 and 3). In the last decade, at the time of arrival in the Netherlands, approximately 60% of these women were aged 20 and over. The largest groups of female migrants were from Somalia, Ethiopia, and Egypt with high FGM/C prevalence and from Ghana and Iraq with low FGM/C prevalence.

It was estimated that about 41,000 female migrants originating from countries where FGM/C is practiced have undergone FGM/C, resulting in a prevalence of 43% (see Table 2). About 15,000 of these 41,000 female migrants have most probably undergone infibulation, most prevalent in the Somalian population. It was also estimated that 4,190 girls are at risk of FGM/C in the near future. Of the girls at risk, approximately 41% were at risk of infibulation. The number of first-generation girls at risk of FGM/C was only 394 (9%), reflecting low immigration of young girls in recent years. Second-generation girls at risk of FGM/C are predominantly from 5 countries, with in descending order Somalia (45%), Egypt (15%), Ethiopia (8%), Sierra Leone (8%), and Guinea (6%). It is predicted that in the next 5 years 1,677 girls are at risk of FGM/C (355 per year), and in the 15 years thereafter another 2,514 girls are at risk of FGM/C (168 per year).

**Table 2:** Estimated numbers of girls and women already undergone FGM/C by Type III, and estimated number of girls at risk of FGM/C.

	Undergone FGM/C			Number of girls at risk of FGM/C			
Country of origin	Prevalence of FGM/C (%)	Total number of girls and women with FGM/C	Type III	First generation	Second generation	Girls at risk of Type III	Total
Benin	6.7	12.42	1.25	0.27	1.41	0.17	1.68
Burkina Faso	56.6	158.50	1.90	0.98	10.70	0.14	11.67
Cameroon	0.8	13.36	0.67	0.30	10.16	0.52	10.46
Central African Rep.	16.6	6.62	0.46	0.13	0.70	0.06	0.83
Chad	24.0	15.82	1.49	0.20	2.89	0.29	3.09
Côte d'Ivoire	26.9	237.22	20.64	2.12	22.21	2.12	24.34
Djibouti	71.6	101.61	68.28	0.56	7.59	5.47	8.14
Egypt	62.1	6168.26	43.18	121.25	555.39	4.74	676.64
Eritrea	57.1	4025.79	1553.96	105.67	67.19	66.72	172.86
Ethiopia	57.9	5899.94	383.50	27.83	305.85	21.69	333.68
Gambia	57.4	363.22	0.00	1.49	10.63	0.00	12.11
Ghana	2.0	243.31	19.22	0.48	15.70	1.28	16.17
Guinea	66.2	1387.48	104.06	3.96	214.46	16.38	218.41
Guinea-Bissau	34.5	63.39	3.80	0.08	6.77	0.41	6.85
Iraq	16.5	1659.91	0.00	14.79	73.55	0.00	88.33
Kenya	33.1	824.87	76.71	28.60	52.82	7.57	81.42
Liberia	34.5	443.79	0.00	1.43	76.54	0.00	77.97
Mali	72.6	128.44	13.61	0.12	1.93	0.22	2.05
Mauritania	44.5	41.86	1.88	0.01	1.69	0.08	1.70
Niger	1.9	2.40	0.15	0.02	0.34	0.02	0.36
Nigeria	24.6	1491.48	79.05	10.08	125.15	7.17	135.23
Senegal	16.4	131.87	9.36	0.74	6.58	0.52	7.31
Sierra Leone	60.8	1410.95	126.99	14.66	295.02	27.87	309.68
Somalia	71.0	14012.37	11111.81	24.21	1722.30	1384.98	1746.51
Sudan	65.1	1959.98	1509.18	32.83	191.95	173.08	224.78
United Rep. of Tanzania	8.1	95.16	6.28	0.86	13.23	0.93	14.10
Togo	3.8	31.28	4.82	0.11	2.58	0.42	2.70
Uganda	0.3	4.05	0.00	0.27	0.55	0.00	0.82
Yemen	11.8	58.66	0.00	0.15	0.30	0.00	0.45
**Total**	**42.9**	**40994.01**	15142.26	394.18	3796.17	1722.84	**4190.35**

The sensitivity analysis showed that changes of 25% in the reduction factors resulted in a 7% downwards to 9% upwards estimation of the FGM/C prevalence among female migrants in the Netherlands. The respective results for girls at risk of FGM/C were a 15% downwards and 17% upwards estimation (see Table 3). The scenario with no impact of prevention activities (0%) or acculturation (0%) had the highest estimates for FGM/C prevalence (24% increase) and girls at risk (35% increase).

**Table 3:** Results of the sensitivity analysis.

Reduction factors			FGM/C prevalence	FGM/C-risk	
Prevention' impact factor (%)	Migration and acculturation' impact factor (%)	Prevention' and 'Migration and acculturation' impact factors combined (%)		First-generation	Second-generation
50	50	75	40994	394	3796
0	0	0	48026	533	4711
25	50	62.5	38213	229	3255
50	25	62.5	39075	394	3273
50	75	87.5	42913	394	4276
75	50	87.5	43775	495	4301
75	75	87.5	44510	495	4276

Dashed boxes indicate base scenario.

## Discussion

In the Netherlands it is estimated that about 41,000 first- and second-generation girls and women on a total of approximately 95,000 girls and women, originating from countries where FGM/C is practiced, have undergone FGM/C. The prevalence of FGM/C in the Netherlands is mainly concentrated among girls and women from six FGM/C practicing countries (i.e., Somalia, Egypt, Ethiopia, Eritrea, Sudan and Iraq). A substantial proportion has suffered from infibulation. About 4,000 girls were estimated at risk of undergoing FGM/C in the next 20 years. Of the girls at risk, about 41% were at risk of infibulation.

These figures have significant implications for targeting girls and women in prevention programs and for identifying medical needs and subsequent provision of health care resources in the Netherlands. Our estimation method showed the importance for validity of these estimates of adjustments for regional variation in FGM/C in countries of origin, and for ‘*migration and acculturation*’ and ‘*preventive measures’* to target girls potentially at risk in country of destination. Further, several methodological choices and particular assumptions have influenced the presented estimation of FGM/C prevalence and girls at risk. Crucial methodological choices pertain to the use of age-specific FGM/C prevalence in 5-year age groups in countries of origin, and adjustments for regional differences in the prevalence of FGM/C in countries of origin.

The customary age for cutting in countries of origin (also referred as the median age for cutting) is often used to distinguish between girls at risk and cut girls. To avoid overestimation, girls whose age is above the customary age for cutting are usually excluded from FGM/C risk calculation (e.g., refs.[11,15]). However, we believe that the use of age-specific FGM/C prevalence- and risk estimation have particular advantages compared to the use of customary age for cutting in our model. The DHS and MICS data on age at which FGM/C is performed, show that in some countries FGM/C is not limited to a narrow age interval. In several countries (i.e., Central African Republic, Guinea-Bissau, Kenya, Liberia, Sierra Leone and the United Republic of Tanzania) between 11 per cent (Central African Republic) and 28 per cent (the United Republic of Tanzania) of cut girls and women underwent the procedure after the age of 15, while the median age for FGM/C is usually lower than 15. Our preference for age-specific figures is in line with existing evidence that in some communities, girls and women are at risk of being cut until they are married or due to other social pressures [11,38]. In addition, the customary age for cutting appears to be less relevant in the migration context, where the ‘opportunity to cut’ might be a more prominent factor determining the practice of FGM/C than the customary age at cutting [11]. Our improved method also avoids the scepticism expressed by Ziyada and colleagues [15] concerning the exclusion of first- and second-generation girls who have been younger than the customary age for FGM/C in their countries of origin (at the time of arrival), but older than that age at time of data collection.

The adjustment for regional variation of FGM/C was conducted in 21 out of 29 countries, illustrating the potential importance of this improvement. However, the influence on the estimated FGM/C prevalence among female migrants in the Netherlands could be limited, since the estimation is largely driven by the countries of origin with FGM/C prevalence well above 80%. In these countries FGM/C is almost universal, with almost no variation in FGM/C prevalence among regions.

Crucial assumptions in the estimation method relate to the magnitude of impact factors for ‘*preventive measures*’ and ‘*migration and acculturation*’ and the introduction of the year 2006 as turning point in FGM/C policy in the Netherlands. To our knowledge, this study is the first to consider the influence of preventive measures to target girls potentially at risk in country of destination while estimating FGM/C. In the Netherlands, no suspected FGM/C case has, as yet, been convicted by a court [33] and there are some studies reporting cultural change in relation to cutting girls [28–32]. However, although these studies have informed our understanding of FGM/C in a new social context, they are restricted to particular FGM/C practising communities. Moreover, the majority of these studies are qualitative in nature, therefore, the findings need to be interpreted with caution. Further, no study has been conducted into the effectiveness of the preventive measures to target girls potentially at risk in the Netherlands. Therefore, in view of the absence of quantitative data to derive empirical reduction factors that capture change in FGM/C practice due to attitude change towards discontinuation of the practice and due to the introduction of the nationwide action plan in the Netherlands, the *‘migration and acculturation impact factor’* and *‘preventive impact factor’* was set at 50%. This represents a balanced scenario (50:50), indicating that 50% of female migrants may have been reached with preventive measures in Netherlands from 2006 onwards, and 50% reduction in FGM/C due to cultural change in views and behaviour concerning cutting of girls after migration. Our sensitivity analysis with different impact factors showed that our estimates are to some degree robust, even if no reduction in FGM/C estimates is applied.

Although our extrapolation model estimated the number of girls and women who have undergone FGM/C and those at risk of being cut, it is difficult to directly compare our findings on the number of girls and women with FGM/C or still at risk for the practice with other Member States in the European Union (EU). This is mainly due to assumptions considered in the model and the availability- and variation in data on female migrants in the EU countries concerned.

In order to have comparable figures across Member States, the European Institute for Gender Equality (EIGE) recommended a common methodological framework to estimate the number of girls and women who have undergone FGM/C and those at risk of being cut in the Member States [11]. Two scenarios of FGM/C risk were defined in the corresponding model in order to determine a risk interval estimation. The high- and the low risk scenarios delimit the boundaries of this interval that take into consideration the influence of migration and acculturation on attitudes and behavior towards cutting girls through ‘migration and acculturation impact factor’. In the EIGE model the national FGM/C prevalence rate of the age cohort 15 to 19 is applied to the total number of first- and second-generation girls who are younger than the customary age for cutting. In 2014, this methodology was used in a pilot study in three Member States [11] leading to further refinements in 2017 and applied to an additional six Member States, Belgium, Greece, France, Italy, Cyprus and Malta [25]. Considering the low- and high-risk scenarios, the number of girls at risk of FGM/C varied between 453 and 748 in Greece and between 24,660 and 44,106 in France. Initially we have adopted this approach to estimate the number of girls at risk of FGM/C in the Netherlands. The number of girls at risk of FGM/C varied between 4970 and 8996. We then refined our model by considering the legal and policy context in terms of preventive measures to target girls potentially at risk in the Netherlands, using age-specific FGM/C prevalence in 5-year age groups in countries of origin and adjusting for regional differences in the prevalence of FGM/C in countries of origin. After these refinements we estimated that in total 4,190 girls were at risk of FGM/C of whom 394 were first-generation girls. Due to these refinements, our estimates are considerably lower than the estimates derived from the methodological framework recommended by EIGE. Despite these refinements, estimating the number of girls and women who have undergone FGM/C and those at risk of FGM/C remain affected by several uncertainties. Hence, these estimations need to be interpreted with some degree of caution, to avoid misuse or misinterpretation of data and stigmatisation of communities affected.

Our estimates underline the importance of prevention strategies to protect girls at risk and to provide appropriate healthcare for those who have undergone FGM/C. It is important to ensure that women with FGM/C are identified and offered appropriate healthcare when necessary. Therefore, capacity building for healthcare professionals, supporting the specialised FGM/C units in the Netherlands [39] and the implementation of guidelines on the management of FGM/C, which are currently being developed by the Dutch Society of Obstetrics and Gynaecology (NVOG), should be priorities in the Netherlands. Furthermore, community engagement is increasingly recognized as crucial both for prevention as well as for raising awareness of, and trust in service [40]. They could also play an important role in awareness raising particularly in recently resettled migrants. Therefore, the strong partnerships in the Netherlands with communities concerned should be supported and maintained. Concern has been expressed in the Netherlands about the cultural competence, training and record keeping of the Youth health care professionals who are explicitly required to identify, assess and record the risk of FGM/C [41]. Good quality record keeping is required and essential in order to evaluate the prevention policies in the Netherlands. Youth health care professionals could also benefit from training on FGM/C to enhance their knowledge, skills, cultural competence- and confidence to appropriately identify and prevent girls from being cut. We suggest further that policy makers and other stakeholders to incorporate systems for monitoring and evaluation into their plans of action in order to evaluate reach and effectiveness.

### Limitations

The current study offers the most comprehensive approach to estimate the number of girls and women who have undergone FGM/C, and the number of girls at risk of FGM/C in the Netherlands. Although the current study is more comprehensive than the previous 2013 study [13], some limitations apply.

In the first place, migrants from Eritrea were until 1993 registered in the Netherlands as originating from Ethiopia. As a consequence, the number of migrants from Eritrea are underreported in the current study. Secondly, unfortunately, we were unable to include undocumented female migrants in our estimations. Currently, data on undocumented migrants are not available and data from unofficial sources provide only proxies. Thirdly, we only included the 29 countries with available nationally representative data on FGM/C. However, there is anecdotal evidence that FGM/C is also practiced in other countries in Asia (e.g., India and Malaysia), Africa (e.g., Congo and Malawi), Eastern Europe (e.g., Dagestan), the Middle East (e.g., Iran and Oman) and South America (e.g. Colombia)[42]. In the absence of (representative survey) data, we were unable to estimate the prevalence and risk of FGM/C among female migrants originating from these countries. Fourth, our extrapolation model fails to consider the process of selection of migrants. In their study on the improvement of FGM/C estimates among migrants in western countries, Ortensi and colleagues [18] stated that on average migrants are usually younger, wealthier and more educated than those in their country of origin. Since lower age and higher levels of wealth- and education or urban residence are usually associated with lower occurrence of FGM/C, the application of the prevalence in the country of origin to female migrants in the country of destination is likely to bias the indirect estimates of FGM/C prevalence. Although we were able to adjust for variation in the FGM/C prevalence among regions in countries of origin and age, unfortunately, due to limited time, we were unable to adjust our figures according to other components of the selection hypothesis.

Nevertheless, while there is association between FGM/C and factors such as education, economic status and urban or rural residence, they need to be interpreted cautiously, since these associations may be due to the confounding effects of ethnicity or other factors [14]. In addition, the influence of education and economic status on the estimated FGM/C prevalence among female migrants in the Netherlands could be limited, since the estimation is driven by countries where the practice is almost universal among the wealthiest and poorest, and among high educated and low educated. In Somalia, for instance, there is no difference in FGM/C prevalence among the poorest (98.4%) and richest (96.2%) wealth quintiles [14] or between women with no education (98%) and secondary education (96.3%) [14].

## Conclusion

In the present study, we aimed to estimate the number of girls and women who have undergone FGM/C before January 1^st^, 2018 and the number of girls at risk of subsequent FGM/C in the next 20 years. Our findings suggest that an estimated 41,000 of the 95,000 women have undergone FGM/C of which 37% had type III, the most severe type. In total 4,190 girls are estimated to be at risk of FGM/C in the next 20 years, of whom 394 are first-generation girls. Those who have undergone the practice may need health care assistance, and those at risk of FGM/C in the near future should be targeted with preventive and protective measures to target girls potentially at risk.

## Supporting information

S1 Appendix Additional information about the data sources used. (DOCX)

S1 Dataset (XLSX)
