# Supplementary material for: The prevalence and risk of Female Genital Mutilation/Cutting among migrant women and girls in the Netherlands: An extrapolation method
Source: PLoS One. 2020 Apr 9;15(4):e0230919. doi: 10.1371/journal.pone.0230919 (PMC7144964; doi:10.1371/journal.pone.0230919)
Supplement: S1 Appendix — (DOCX) [file pone.0230919.s001.docx]

**Information about the data sources used**

*FGM/C typology*

Data on FGM/C typologies are not available for Iraq, Liberia and Uganda. We assumed a zero prevalence of Type III in these three countries. Data on FGM/C typologies for Egypt are from DHS 1995 and data for Eritrea are from DHS 2002 since more recent typologies data are unavailable.

*Age at cutting*

Using data on age at cutting, we aimed to compute FGM/C prevalence for the age group under 15. We therefore used data on age at which FGM/C is performed, derived from respondents between 15 and 49 years in DHS and MICS surveys. These data were first adjusted for women with missing information on age at cutting, and subsequently age-specific proportions were multiplied with the national FGM/C prevalence for age group 15 to 19 years. However, data on age at cutting for girls and women aged 15 to 49 are unavailable for Benin (MICS 2014), Central African Republic (MICS 2010), Ghana (MICS 2010) and Guinea-Bissau (MICS 2014). For these countries we instead used (adjusted) data on age at cutting for daughters (0 to 14) from most recent reports available.

Data on age at cutting are unavailable for Cameroon (DHS 2004), Djibouti (MICS 2006), Sudan (2014) and Iraq (MICS 2011). For these countries we instead extracted data from country profile UNICEF in combination with most recently available DHS and MICS FGM/C prevalence rate for the age cohort 15 to 19.

*Data on migrant population in the Netherlands*

Number of second-generation girls and women from (the Kurdish Autonomous Region of) Iraq were estimated as Statistics Netherlands (CBS) does not provide information regarding the second-generation girls and women from (the Kurdish Autonomous Region of) Iraq.
